# Secular trends in ESBL, MRSA and VRE incidence in German intensive care units

**DOI:** 10.1186/1753-6561-5-S6-O4

**Published:** 2011-06-29

**Authors:** R Leistner, S Hansen, F Schwab, C Geffers, E Meyer, P Gastmeier

**Affiliations:** 1 Institute of Hygiene and Environmental Medicine, Charite, Berlin, Germany; 2 German National Reference Centre for Surveillance of Nosocomial Infections, Berlin, Germany

## Introduction / objectives

Aim of our study was to quantify and compare trends in incidence of ESBL producing bacteria, MRSA and VRE in intensive care units participating in the German Nosocomial Infection Surveillance System (ICU-KISS) over the last five years.

## Methods

A surveillance module for multidrug resistant bacteria (MDR-KISS) was added to ICU-KISS in 2006. Participating ICUs report data on patients with MRSA, VRE and ESBL bacteria*.* In contrast to ICU-KISS on nosocomial infections, MDR-KISS documents cases of MDR bacteria including colonization and infection present on admission. MDR-KISS collects the following data: date of ICU-admission and ICU-discharge, type of MDR-bacteria and date of first detection, presence of MDR-bacteria on admission or acquisition during the ICU-stay. Incidence of these bacteria was calculated per 100 patients and density per 1000 patient-days.

## Results

Up to 2010, 325 ICUs reported data on 253,756 patients. The incidence of ESBL tripled over the last five years from 0.2 to 0.7 per 100 patients. 34% of the ESBL infections were hospital acquired. MRSA incidence stayed stable over time with 1.5 per 100 patients. The incidence of hospital acquired MRSA decreased from 25% in 2006 to 18% in 2010. VRE incidence ranged between 0.1 and 0.2. Hospital acquired VRE showed an incidence of 57% in 2006 and 55% in 2010.

## Conclusion

ESBL incidence has been rising steadily and the majority of ESBL producing bacteria were not acquired in the ICU. The dynamic of the ESBL resistance runs not parallel to the stable resistance situation in Gram-positives (MRSA or VRE). To improve the resistance situation it might not be sufficient to simply restrict interventions to ICUs. Hence, not only ambulatory health care but also veterinary medicine should be included. Physicians and veterinaries should be urged to use antibiotics prudentially for humans and animals.

## Disclosure of interest

None declared.

